# P25 Pharmacovigilance assessment of Stevens–Johnson syndrome/toxic epidermal necrolysis (SJS/TEN) with antibacterial drugs using FDA adverse event reporting system data

**DOI:** 10.1093/jacamr/dlae136.029

**Published:** 2024-08-23

**Authors:** Sriram Radhakrishnan, Vaishnavi Velmani, K Shailaja

**Affiliations:** Department of Pharmacy Practice, C.L. Baid Metha College of Pharmacy, Affiliated to the TamilNadu Dr.MGR Medical University, Chennai-97, India; Department of Pharmacy Practice, C.L. Baid Metha College of Pharmacy, Affiliated to the TamilNadu Dr.MGR Medical University, Chennai-97, India; Department of Pharmacy Practice, C.L. Baid Metha College of Pharmacy, Affiliated to the TamilNadu Dr.MGR Medical University, Chennai-97, India

## Abstract

**Background:**

Stevens–Johnson syndrome (SJS) and toxic epidermal necrolysis (TEN) are severe and potentially life-threatening skin reactions associated with certain medications, including antibacterial drugs. This study outlines a comprehensive pharmacovigilance study using the US FDA Adverse Event Reporting System (FAERS) data to assess this association.

**Methods:**

All SJS/TEN cases of antibacterial drugs as primary suspected drugs were extracted from the US FDA FAERS from 2004 to 2023. Descriptive analyses were performed to evaluate the prevalence of SJS/TEN among antibacterial drug users. Disproportionate analyses were conducted by estimating the reporting odds ratio (ROR) and the information component (IC).

**Results:**

Out of a total of 38 185 cases of SJS/TEN reported in the FAERS, 16 104 (42.17%) cases are due to antibacterial drug use suspects. 332 cases of SJS/TEN overlap from the year 2019 reported. Gender analysis of SJS/TEN shows females (51.2%) are more prone than males (39.29%) and not specified (9.51%). Mortality rate of SJS/TEN due to drug adverse reaction is 21% (7995). The SJS/TEN signal was detected majorly in cotrimoxazole, amoxicillin, ciprofloxacin, vancomycin, levofloxacin, piperacillin sodium/tazobactam sodium, amoxicillin/clavulanate potassium and clarithromycin. The ROR (95% CI) of cotrimoxazole, amoxicillin, ciprofloxacin, vancomycin, levofloxacin, piperacillin sodium/tazobactam sodium, amoxicillin/clavulanate potassium and clarithromycin were 53.74 (57.62–50.12), 0.67 (0.72–0.63), 0.54 (0.58–0.50), 0.85 (0.91–0.79), 0.30 (0.32–0.27), 0.89 (0.96–0.82), 11.27 (12.39–10.25) and 0.50, (0.55–0.46), respectively.

**Conclusions:**

This pharmacovigilance assessment of FAERS shows 42.17% association of antibacterial drugs in suspected cases of SJS/TEN. This reminds the medical workers to pay attention to the severe adverse effects of antibacterial drugs leading to SJS/TEN.

